# WSSV-induced reversal of the malate-aspartate shuttle facilitates viral replication in shrimp hemocytes

**DOI:** 10.1186/s12964-025-02506-3

**Published:** 2025-11-26

**Authors:** Fang-Jyun Guo, Kuan-Lun Huang, Cong-Yan Chen, Shu-Wen Cheng, Yen Siong Ng, Han-Ching Wang

**Affiliations:** 1 Department of Biotechnology and Bioindustry Sciences, College of Biosciences and Biotechnology, National Cheng Kung University, Tainan, Taiwan; 2 International Center for the Scientific Development of Shrimp Aquaculture, National Cheng Kung University, Tainan, Taiwan

**Keywords:** *Litopenaeus vannamei*, White spot syndrome virus, Malate-aspartate shuttle, Malate dehydrogenase, Glutamate-oxaloacetate transaminase, Oxoglutarate carrier, Aspartate-glutamate carrier

## Abstract

**Background:**

White spot syndrome virus (WSSV), one of the most devastating pathogens in global shrimp aquaculture, has been shown to hijack and reprogram host metabolic pathways to support its replication. Among the various host metabolic circuits, the malate-aspartate shuttle (MAS) is a key redox-balancing mechanism that facilitates the translocation of cytosolic NADH into mitochondria, thereby sustaining glycolysis and mitochondrial function. To date, however, the involvement of MAS in WSSV pathogenesis has not been documented.

**Methods:**

In this study, we investigated the role of the MAS pathway in WSSV replication. We first assessed the mRNA level changes of MAS-related genes in WSSV infected shrimp. dsRNA-mediated gene silencing was also employed to examine its impact on the virus replication. To determine the direction of MAS during WSSV infection, we first silenced the MAS-related genes (e.g., GOT1 or GOT2), and subsequently replenished the corresponding metabolite to assess whether it could rescue the virus replication.

**Results:**

At the viral genome replication stage of WSSV infection (12 hpi), significant upregulation of key MAS-related genes, including *Lv*GOT1, *Lv*GOT2, *Lv*MDH1, *Lv*AGC, and *Lv*OGC, was observed in hemocytes of infected shrimp. Functional knockdown of these genes by *in vivo* dsRNA-mediated gene silencing significantly reduced WSSV gene expression and viral genome copy number, indicating that MAS activity is required for efficient WSSV replication. Furthermore, metabolite rescue experiments revealed a potential reversal of the MAS flux during the infection: supplementation with aspartate or α-ketoglutarate restored viral replication in *Lv*GOT1-silenced shrimp, while oxaloacetate supplementation reversed the lowered replication caused by *Lv*MDH1 silencing.

**Conclusion:**

This study demonstrates that WSSV activates the host MAS pathway to facilitate its replication and highlights the dynamic reprogramming of redox-associated mitochondrial metabolism in response to WSSV infection. The WSSV-induced reversed MAS might supply specific metabolites such as aspartate needed for virus replication or to prevent the TCA shut down.

**Supplementary Information:**

The online version contains supplementary material available at 10.1186/s12964-025-02506-3.

## Background

**Fig. 1:**
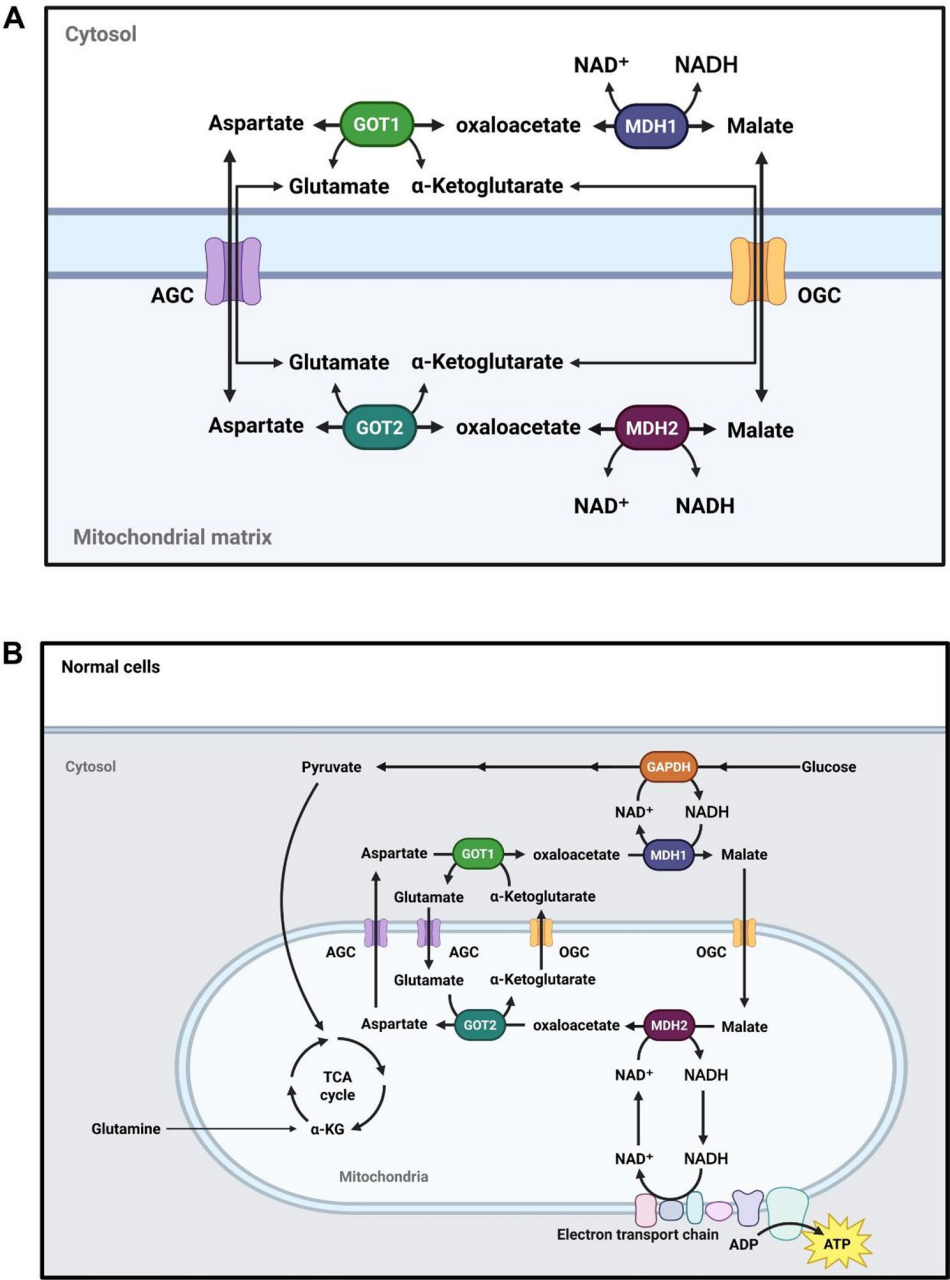
The malate-aspartate shuttle. **A** Schematic diagram showing the components of the malate-aspartate shuttle (MAS) and their localizations in the cell. The malate-aspartate shuttle is composed of cytosolic GOT1 and MDH1, mitochondrial GOT2 and MDH2, and two transport proteins AGC and OGC. The catalytic reactions of both GOTs and MDHs are reversible. **B** MAS in normal cellular metabolism. Figs. 1A and B were adapted and modified from Broeks et al. (2021) [6] and Broeks et al. (2023). GOT1/2, glutamate-oxaloacetate transaminase 1/2. MDH1/2, malate dehydrogenase 1/2. AGC, aspartate-glutamate carrier. OGC, 2-oxoglutarate carrier

Nicotinamide adenine dinucleotide (NAD) is a fundamental redox coenzyme that exists in two interconvertible forms: the oxidized form (NAD⁺) and the reduced form (NADH). It plays a pivotal role as an electron carrier in numerous biochemical reactions, particularly those involving energy metabolism [1, 2]. In catabolic pathways such as glycolysis, glutamine metabolism (glutaminolysis), the tricarboxylic acid (TCA) cycle, and fatty acid β-oxidation, NAD⁺ serves as an essential electron acceptor for dehydrogenase-mediated reactions and is concomitantly reduced to NADH. The resulting NADH subsequently donates electrons to the mitochondrial electron transport chain (ETC), located on the inner mitochondrial membrane, thereby driving ATP production through oxidative phosphorylation [1, 3, 4]. Since NADH cannot directly cross the inner mitochondrial membrane, cells employ unique mechanisms, including the malate-aspartate shuttle (MAS) and the glycerol phosphate shuttle, to transfer cytosolic reducing equivalent NADH into mitochondria [5]. In the malate-aspartate shuttle (MAS), cytosolic malate dehydrogenase 1 (MDH1) converts oxaloacetate (OAA) to malate and oxidizes NADH to NAD⁺. Malate is then transported into mitochondria via the oxoglutarate carrier (OGC) and converted back to OAA by mitochondrial MDH2, coupled with the reduction of NAD⁺ to NADH. OAA is subsequently converted to aspartate by glutamate-oxaloacetate transaminase 2 (GOT2) and transported to the cytosol via the aspartate-glutamate carrier (AGC). Cytosolic GOT1 converts aspartate back to OAA, completing the cycle (Fig. 1B) [5]. The malate-aspartate shuttle functions as a “bridge,” connecting metabolic pathways between cytosol and mitochondria by transferring metabolites and reducing equivalent NADH. MDH1 reoxidizes cytosolic NADH, which is primarily generated by the glycolytic enzyme glyceraldehyde 3-phosphate dehydrogenase (GAPDH), to NAD⁺, thereby maintaining cellular redox homeostasis and supporting glycolysis [1, 6]. Mitochondrial MDH2 and GOT2 reduce NAD⁺ to NADH and produce TCA cycle intermediates, including oxaloacetate and α-ketoglutarate (α-KG). The resulting NADH enters the electron transport chain to drive ATP synthesis [6–8]. Furthermore, GOT1 and GOT2 also play key roles in glutamine-driven anaplerosis, with the produced aspartate contributing to nucleotide and amino acid biosynthesis [9].

In colorectal cancer (CRC), breast cancer, and pancreatic ductal adenocarcinoma (PDAC), cells show high dependency on the non-canonical glutamine metabolic pathway [11]. In this pathway, GOT1 converts aspartate (derived from mitochondrial GOT2) into cytosolic oxaloacetate, which supports the subsequent reactions mediated by MDH1 and malic enzyme 1 (ME1) to generate NADPH that can be used to resist reactive oxygen species (ROS). Targeting GOT1 or disruption of this pathway has been reported to cause a decrease in the NAD^+^/NADH ratio, increase in the NADP^+^/NADPH ratio and reduced ATP production, collectively contributing to suppression of cancer cell proliferation and enhancement of sensitivity to anti-tumor therapies [12–15]. In pancreatic cancer, loss of GOT2 causes dysfunction of the malate-aspartate shuttle and subsequently disrupts cellular redox homeostasis while impairing both the TCA cycle and glycolysis [16]. Another study reported that SIRT3 negatively regulates the activity of the malate-aspartate shuttle by deacetylating GOT2. Low SIRT3 expression level was observed in human pancreatic tumor with increased GOT2 acetylation, and this enhances its interaction with MDH2, thereby promoting the transfer of NADH from cytosol to mitochondria via MAS. This process facilitates the production of ATP and NADPH, while suppressing ROS [17]. MDH2 is highly expressed in prostate cancer cells. Silencing MDH2 disrupts the TCA cycle, accompanied by an increase in the mitochondrial NAD⁺/NADH ratio and the intracellular ADP/ATP ratio, indicating impaired energy metabolism. This metabolic disorder enhances the sensitivity of prostate cancer cells to docetaxel treatment [18]. Furthermore, under glutamine-limited conditions, AGC-mediated transport of aspartate from mitochondria to cytosol is critical for cell proliferation and survival. Knockdown of AGC decreases cytosolic aspartate levels and the NAD⁺/NADH ratio, thereby promoting a shift toward the oxidative TCA cycle and increasing cellular dependence on glutamine [19]. In another study focusing on OGC in non-small cell lung cancer (NSCLC) and melanoma, loss of OGC impairs MAS function by blocking the transport of cytosolic NADH into mitochondria. This disruption suppressed oxidative phosphorylation and ATP production, leading to a reduction in TCA cycle intermediates and decreased oxygen consumption [20].

In cases of virus infection, *Siniperca chuatsi* rhabdovirus (SCRV) and infectious spleen and kidney necrosis virus (ISKNV) have been shown to upregulate the expression of GOT1, GOT2, MDH1 and MDH2, thereby supplying substrates for asparagine biosynthesis and promoting virus replication [21, 22]. Kaposi’s sarcoma-associated herpesvirus (KSHV) upregulates GOT2 expression to accelerate glutamine metabolism, providing a nitrogen source for purine and pyrimidine biosynthesis [23, 24]. Gallid Alpha-Herpesvirus 1 (also known as avian infectious laryngotracheitis virus, ILTV) exploits Fos transcriptional factor to regulate MDH1, thereby supplying OAA to the TCA cycle and promoting virus replication [25]. Moreover, treatment with the MAS inhibitor aminooxyacetic acid (AOAA) has been shown to suppress viral replication [21–23]. Taken together, these findings highlight the importance of MAS components in cancer cell proliferation and virus replication.

White spot disease (WSD) is one of the deadliest viral diseases in shrimp farming. The causative agent, white spot syndrome virus (WSSV) has been demonstrated to induce metabolic reprogramming in host cells to facilitate WSSV replication [26]. Previous studies have reported that during the viral genome replication stage (12 h post WSSV injection, 12 hpi), WSSV activates aerobic glycolysis (also known as the Warburg effect) to enhance ATP production and provide carbon backbones for biosynthesis, while glucose-derived intermediates are funneled into the pentose phosphate pathway (PPP) to promote nucleotide biosynthesis [27–30]. At 6 and 12 hpi, significant increases in the NADH/NAD^+^ ratio resulted from activation of the glycolytic pathway and subsequent NADH accumulation in the early stage of WSSV infection [31]. Additionally, WSSV infection also induces glutamine metabolism during the viral genome replication stage to utilize glutamine (or glutamate) as an alternative carbon source for replenishing the TCA cycle. Stable-isotope labeled glutamine tracking has revealed that both oxidative glutamine metabolism and reductive carboxylation are triggered, with significant upregulation in the mRNA expression and activity of glutamine metabolism-related enzymes including glutaminase (*Lv*GLS), glutamate dehydrogenase (*Lv*GDH), isocitrate dehydrogenase 1/2 (*Lv*IDH1/2) and α-ketoglutarate dehydrogenase (*Lv*α-KGDH) [32, 33]. However, the specific roles of the MAS-related enzymes *Lv*GOT1 and *Lv*GOT2 in WSSV-induced glutamine metabolism remain to be elucidated. Furthermore, at the late stage of WSSV infection, the loss of mitochondrial membrane potential and the decrease in ATP production suggests dysfunction of the electron transport chain [28]. Whether this impairment is associated with dysregulation of the malate-aspartate shuttle and the consequent disruption of cellular redox homeostasis still requires further investigation. Although several studies have examined the roles of GOT and MDH in glutamine metabolism and the TCA cycle, respectively, during viral replication, their functions as components of MAS remain unexplored in virus replication, particularly in WSSV. Our study therefore aims to explore the role of these MAS components in WSSV-induced metabolic reprogramming.

In this study, we first analyzed the changes in mRNA expression of MAS-related genes, including *Lv*GOT1, *Lv*GOT2, *Lv*MDH1, *Lv*MDH2, *Lv*AGC and *Lv*OGC, during WSSV infection. Next, *in vivo* dsRNA-mediated gene silencing verified the importance of the malate-aspartate shuttle in WSSV replication. Additionally, metabolite replenishment after silencing MAS-related gene was conducted to investigate the direction of MAS flux during WSSV infection. Our findings were used to evaluate the critical role of malate-aspartate shuttle in WSSV replication and provide an initial insight into how WSSV might manipulate the host malate-aspartate shuttle to benefit its replication.

## Methods

### Experimental animals and WSSV inoculum

The Pacific white shrimp (*Litopenaeus vannamei*, mean body weight ~ 3 g) used in this study were obtained from the Department of Aquaculture, National Pingtung University of Science and Technology (NPUST), Taiwan. Prior to experimentation, shrimp were maintained in sterilized seawater for 24 h (salinity 20% & temperature 27 °C) to allow acclimatization. The WSSV inoculum (Taiwan isolate; GenBank accession no. AF440570) was prepared from the hemolymph of specific pathogen-free (SPF) white shrimp infected by WSSV. For viral challenge, shrimp were intramuscularly injected at the junction between the first and second abdominal segments with 100 µl of either WSSV inoculum (1:10,000 dilution of viral stock in 1x phosphate-buffered saline [PBS]; 137 mM NaCl, 2.7 mM KCl, 10 mM Na₂HPO₄, 2 mM KH₂PO₄) or PBS alone as a solvent control. The chosen WSSV dose was sufficient to induce approximately 50% cumulative mortality within 3 days and complete mortality by 5 days post-infection. At 12 and 24 h post-injection (hpi), hemolymph was collected to isolate hemocytes for mRNA quantification, and pleopods were sampled to determine WSSV genome copy number via quantitative PCR. For hemocyte collection, hemolymph was withdrawn from beneath the shrimp carapace using a 26G syringe and immediately mixed with an equal volume of anticoagulant (450 mM NaCl, 10 mM KCl, 10 mM EDTA, 10 mM Tris [pH 7.4]). Hemocytes were separated by centrifugation at 10,000 × g for 1 min at 4 °C. The resulting pellet was washed once with 1× PBS and resuspended in 100 µL of PBS. Subsequently, 1 mL of REzol reagent (Protech Enterprise) was added, and the samples were stored at − 80 °C for RNA extraction.

### *In vitro* dsRNA synthesis

To synthesize the double-stranded RNA (dsRNA) used for *in vivo* dsRNA-mediated gene silencing, primer sets for MAS-related genes were designed based on the cDNA sequences of our in-house *L. vannamei* transcriptome database (Table 1). Partial fragments of MAS-related genes and luciferase were amplified using the corresponding Gene-dsF/Gene-dsR primer set. The T7 promoter was then incorporated into the 5’ end of amplicons by PCR using following primer set: T7-Gene-dsF/Gene-dsR & Gene-dsF/T7-Gene-dsR. The final amplicons served as templates for single-strand RNA (ssRNA) synthesis by using T7 RiboMax™ express large scale RNA production system kit (Promega). Specific dsRNA was synthesized by incubating two complementary ssRNAs, followed by purification by phenol/chloroform extraction. The dsRNA products were checked with agarose gel electrophoresis, quantified using a UV Nano-200 Micro-spectrophotometer (Allsheng Instruments, Taiwan), and stored at −80 °C.

**Table 1:** Primers used in this study

Gene^a^	Primer name	Sequence(5’→3’)^b^	Application
*Lv*GOT1 (PVHP186439.4)	*Lv*GOT1-dsF	TTAACCTGAGTGTTGGAGC	dsRNA synthesis
	*Lv*GOT1-dsR	CTTGGTAAGCAGAGTCAA	dsRNA synthesis
	T7-*Lv*GOT1-dsF	*TAATACGACTCACTATAGGGAGA*TTAACCTGAGTGTTGGAGC	dsRNA synthesis
	T7-*Lv*GOT1-dsR	*TAATACGACTCACTATAGGGAGA*CTTGGTAAGCAGAGTCAA	dsRNA synthesis
	*Lv*GOT1-qF	GGTGAGGAAGGTAGAGGCAGAA	Real-time PCR
	*Lv*GOT1-qR	GGCAGGTACTCGTGGTTGAGA	Real-time PCR
*Lv*GOT2 (PVHP161430.1)	*Lv*GOT2-dsF	CTAGTTCTTGGTGGTCTG	dsRNA synthesis
	*Lv*GOT2-dsR	GTGAGCACAGGCGTGAAGC	dsRNA synthesis
	T7-*Lv*GOT2-dsF	*TAATACGACTCACTATAGGGAGA*CTAGTTCTTGGTGGTCTG	dsRNA synthesis
	T7-*Lv*GOT2-dsR	*TAATACGACTCACTATAGGGAGA*GTGAGCACAGGCGTGAAGC	dsRNA synthesis
	*Lv*GOT2-qF	CGGCTCCACCTTCCTCTCA	Real-time PCR
	*Lv*GOT2-qR	GGTGCTGGCAGCCATACAT	Real-time PCR
*Lv*MDH1 (PVHP13035.1)	*Lv*MDH1-dsF	ATCTTCAAGACTCAGGGCC	dsRNA synthesis
	*Lv*MDH1-dsR	ATGGTGATCGGGAAGGAGTACA	dsRNA synthesis
	T7-*Lv*MDH1-dsF	*TAATACGACTCACTATAGGGAGA*ATCTTCAAGACTCAGGGCC	dsRNA synthesis
	T7-*Lv*MDH1-dsR	*TAATACGACTCACTATAGGGAGA*ATGGTGATCGGGAAGGAGTACA	dsRNA synthesis
	*Lv*MDH1-qF	TCTTCTCCGACGGTTCCTACA	Real-time PCR
	*Lv*MDH1-qR	ATGGTGATCGGGAAGGAGTACA	Real-time PCR
*Lv*MDH2 (PVHP178520.1)	*Lv*MDH2-dsF	TCACCGGCTTCGTTGGA	dsRNA synthesis
	*Lv*MDH2-dsR	CATAGGCACATTCCACCACACC	dsRNA synthesis
	T7-*Lv*MDH2-dsF	*TAATACGACTCACTATAGGGAGA*TCACCGGCTTCGTTGGA	dsRNA synthesis
	T7-*Lv*MDH2-dsR	*TAATACGACTCACTATAGGGAGA*CATAGGCACATTCCACCACACC	dsRNA synthesis
	*Lv*MDH2-qF	TCACCGGCTTCGTTGGA	Real-time PCR
	*Lv*MDH2-qR	ACAACTTCACATCCCTTCAGAGAAT	Real-time PCR
*Lv*AGC (PVHP158701.1)	*Lv*AGC-dsF	CTCTGTGTGCCTGATGCTCTCT	dsRNA synthesis
	*Lv*AGC-dsR	TCAGGTGGAAGAGAATCTCCAC	dsRNA synthesis
	T7-*Lv*AGC-dsF	*TAATACGACTCACTATAGGGAGA*CTCTGTGTGCCTGATGCTCTCT	dsRNA synthesis
	T7-*Lv*AGC-dsR	*TAATACGACTCACTATAGGGAGA*TCAGGTGGAAGAGAATCTCCAC	dsRNA synthesis
	*Lv*AGC-qF	CTCTGTGTGCCTGATGCTCTCT	Real-time PCR
	*Lv*AGC-qR	CATTCCAGTTCCCTTAGTATCAAACA	Real-time PCR
*Lv*OGC (PVHP163694.1)	*Lv*OGC-dsF	TGCAGAGGTGTCCCTCATT	dsRNA synthesis
	*Lv*OGC-dsR	CGGAATCCCCTAATACATGCTT	dsRNA synthesis
	T7-*Lv*OGC-dsF	*TAATACGACTCACTATAGGGAGA*TGCAGAGGTGTCCCTCATT	dsRNA synthesis
	T7-*Lv*OGC-dsR	*TAATACGACTCACTATAGGGAGA*CGGAATCCCCTAATACATGCTT	dsRNA synthesis
	*Lv*OGC-qF	CCACACCGTCCTCACTTTCA	Real-time PCR
	*Lv*OGC-qR	CGGAATCCCCTAATACATGCTT	Real-time PCR
Luciferase	Luc-dsF	CTGAATACAAATCACAGAATC	dsRNA synthesis
	Luc-dsR	GCGAGAATCTGACGCAGGCAGT	dsRNA synthesis
	T7-Luc-dsF	*TAATACGACTCACTATAGGGAGA*CTGAATACAAATCACAGAATC	dsRNA synthesis
	T7-Luc-dsR	*TAATACGACTCACTATAGGGAGA*GCGAGAATCTGACGCAGGCAGT	dsRNA synthesis
*Lv*EF1α	*Lv*EF1α-qF	ACGTGTCCGTGAAGGATCTGAA	Real-time PCR
	*Lv*EF1α-qR	TCCTTGGCAGGGTCGTTCTT	Real-time PCR
WSSV VP28	VP28-qF	AGTTGGCACCTTTGTGTGTGGTA	Real-time PCR
	VP28-qR	TTTCCACCGGCGGTAGCT	Real-time PCR
	Anchor-dTv	GACCACGCGTATCGATGTCGACTTTTTTTTTTTTTTTTV	cDNA synthesis

^a^The primer sets for the MAS-related genes were designed using an in-house transcriptomic database. The gene accession number is given below in parentheses

^b^The added T7 promoter sequence is shown in italics

### *In vivo* dsRNA-mediated gene silencing of genes involved in the malate-aspartate shuttle

Shrimp (*n* = 30 for each group) were intramuscularly injected with 100 µl PBS, luciferase dsRNA or target gene dsRNA (diluted with 1x PBS) 72 h before WSSV challenge. The dosage of injected dsRNA was 1 µg per gram shrimp. PBS was used as the solvent control and luciferase dsRNA served as the non-specific silencing control. At 72 h post dsRNA injection, shrimp hemocytes were collected to examine the efficiency of gene silencing using qPCR, and the remaining shrimp were infected with WSSV. Hemocytes were collected again at 12 hpi and 24 hpi for quantification of host or WSSV gene expression. Shrimp pleopods were collected to quantify WSSV genome copy number.

### Quantification of host and WSSV gene expression

Hemocytes (4 pooled samples, 3 shrimp per pool) collected at 12 and 24 hpi were subjected to RNA extraction by using REzol (Protech Enterprise) and reverse transcription-PCR by using SuperScript™ II Reverse Transcriptase (Invitrogen) and Anchor-dTv primer (Table 1). 20x diluted cDNA samples were used to quantify gene expression. mRNA expression of host and WSSV genes were measured by real-time PCR with KAPA SYBR FAST qPCR Master Mix (KAPA) and CFX Connect^™^ Real-Time PCR Detection System (Bio-Rad). Target genes included the host genes *Lv*GOT1, *Lv*GOT2, *Lv*MDH1, *Lv*MDH2, *Lv*AGC, *Lv*OGC and the housekeeping gene *Lv*EF1α (elongation factor 1 alpha) as well as the WSSV structural protein late gene VP28. Primer sets used for real-time PCR are listed in Table 1. In subsequent analysis, the value of *Lv*EF1α served as the internal control for data normalization, and the 2^-∆Ct^ method was used for calculation. Statistical analysis was done as described below.

### Quantification of WSSV genome copy number

Pleopods (4 pooled samples, 3 shrimp per pool) collected at 24 hpi were subjected to DNA extraction by using the DTAB/CTAB DNA extraction kit (GeneReach Biotechnology Corp.) and WSSV genome copy number was quantified by using an IQ Real™ WSSV quantitative system (GeneReach Biotechnology Corp.) according to the manufacturer’s instruction. Statistical analysis was done as described below.

### *In vivo* metabolite replenishment after gene silencing

Metabolite replenishment was conducted as described by Li et al. (2016) [33], Briefly, dsRNAs were synthesized using the method described above and applied for *in vivo* gene silencing. WSSV challenge was conducted at 3 days post dsRNA treatment, followed by 100 µl PBS or metabolite injection at 2 hpi. Metabolites were dissolved in PBS and diluted to the required dose for supplementation. The initial aspartate (Sigma-Aldrich) dosage was set at 500 µg per gram of shrimp, based on Sacchi et al. (2017) [34]. However, this concentration was found to be lethal to shrimp. Therefore, the dosage was subsequently reduced to 15.6 µg per gram shrimp, representing a 32-fold dilution of the original dose; the dosage of α-ketoglutarate (Sigma-Aldrich) was 530 µg per gram shrimp [33]; the dosage of oxaloacetate (Sigma-Aldrich) was 23.4 µg per gram shrimp [35]. 3 g shrimp were used for aspartate & oxaloacetate replenishment experiment, whereas 1.5 g shrimp were used for α-ketoglutarate replenishment experiment. Shrimp hemocytes were collected at 72 h post dsRNA injection, and at 12 hpi and 24 hpi for quantification of host or WSSV gene expression. Shrimp pleopods were collected at 24 hpi for quantification of the WSSV genome copy number. Subsequent sample processing and data analysis were conducted as described above. The silencing efficiency of MAS-related genes was included in Figs. S1 and S2 (See Additional file 1).

### Statistical analysis

Data calculations and graph generation were performed in Microsoft Excel and GraphPad Prism 10 respectively. The Empirical Rule, as described in Tseng et al. (2019) [36], was applied to identify and exclude statistical outliers. Statistically significant differences between groups were evaluated using Student’s *t*-test for multiple treatment comparisons. Data are presented as mean ± SD. Significance is indicated by *p < 0.05, **p < 0.01.

## Results

### WSSV infection induces the expression of MAS-related genes in shrimp

**Fig. 2:**
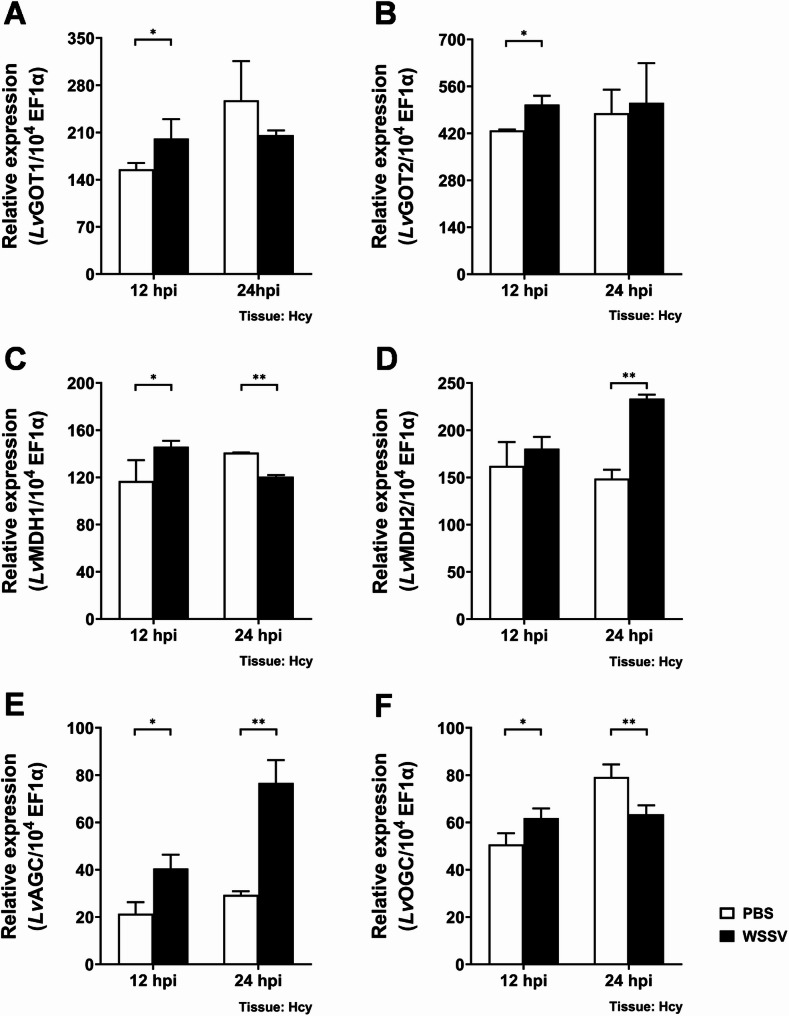
mRNA expression levels of genes involved in the malate-aspartate shuttle during WSSV infection. **A-F** mRNA expression levels of *Lv*GOT1, *Lv*GOT2, *Lv*MDH1, *Lv*MDH2, *Lv*AGC and *Lv*OGC in shrimp hemocytes during WSSV infection. mRNA expression levels were analyzed by real-time PCR. Each bar represents the mean ± SD. Asterisks indicate statistically significant differences between the WSSV-infected group and the corresponding PBS control group (*p < 0.05; **p < 0.01). hpi: hours post PBS/WSSV injection. Hcy: hemocytes

The malate-aspartate shuttle requires the coordination of several proteins, including four catalytic enzymes (GOT1/2 & MDH1/2) and two transport proteins (AGC & OGC), to transfer cytosolic reducing equivalent NADH into the mitochondria (Fig. 1). To understand the effects of WSSV infection on the malate-aspartate shuttle, we first examined the mRNA expression levels of MAS-related genes in WSSV-infected shrimp from 12 to 24 h post WSSV injection (hpi). At 12 hpi, mRNA expression levels of most genes, including *Lv*GOT1, *Lv*GOT2, *Lv*MDH1, *Lv*AGC and *Lv*OGC were significantly increased in the WSSV infected group, as compared to the PBS solvent control group (Fig. 2). However, at 24 hpi, WSSV infection led to an increase in *Lv*MDH2 and *Lv*AGC expression, and a decrease in *Lv*OGC and *Lv*MDH1 expression.

### The malate-aspartate shuttle is crucial for WSSV replication

**Fig. 3:**
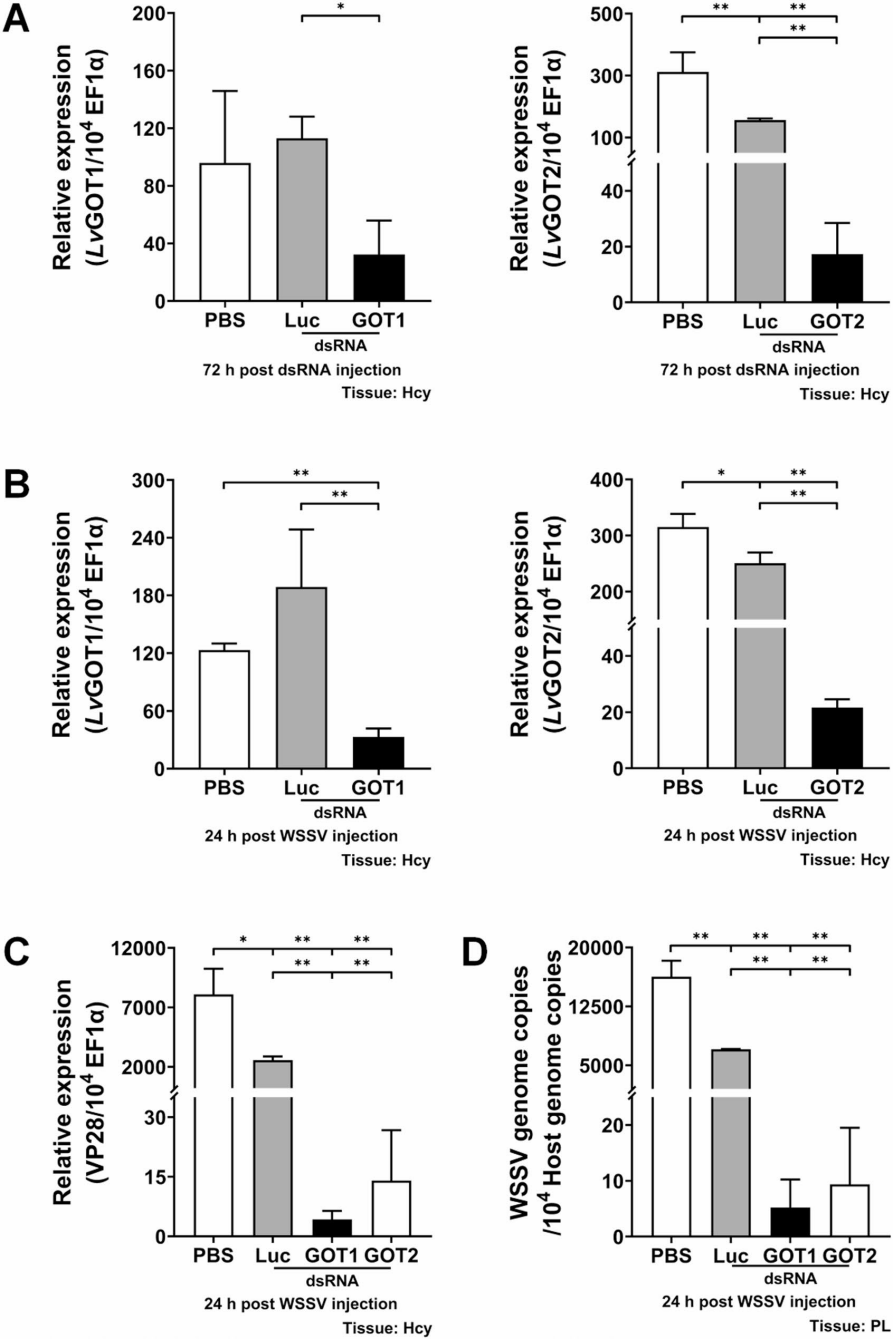
Effects of *Lv*GOTs silencing on WSSV replication. **A** mRNA expression levels of *Lv*GOT1 and *Lv*GOT2 in shrimp hemocytes at 72 h post dsRNA injection and before WSSV injection. **B** mRNA expression levels of *Lv*GOT1 and *Lv*GOT2 in hemocytes at 24 h post WSSV injection. **C** mRNA expression level of WSSV late gene VP28 in hemocytes at 24 h post WSSV injection. **D** WSSV genome copy number was determined in shrimp pleopods at 24 h post WSSV injection. Shrimp treated with PBS or non-specific luciferase (Luc) dsRNA were used as control groups. Each bar represents the mean ± SD. Asterisks indicate statistically significant differences between the control group and *Lv*GOT1 or *Lv*GOT2 dsRNA treated groups (*p < 0.05; **p < 0.01). Hcy: hemocytes. PL: pleopods

**Fig. 4:**
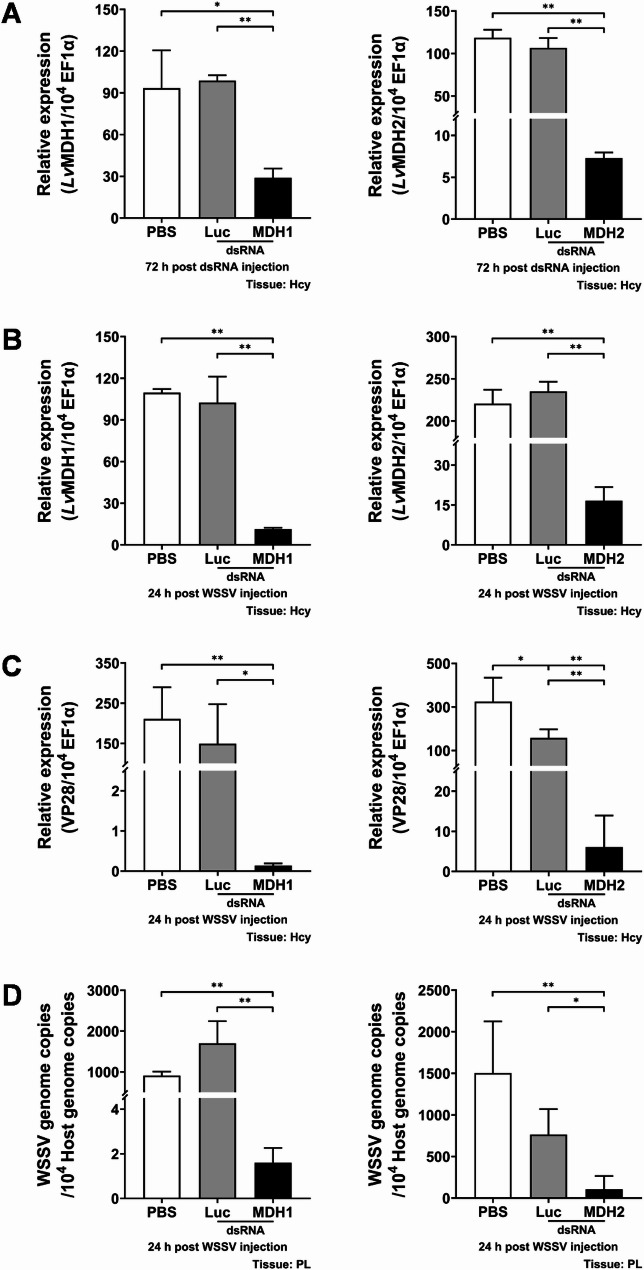
Effects of *Lv*MDHs silencing on WSSV replication. **A** mRNA expression levels of *Lv*MDH1 and *Lv*MDH2 in shrimp hemocytes at 72 h post dsRNA injection and before WSSV injection. **B** mRNA expression levels of *Lv*MDH1 and *Lv*MDH2 in hemocytes at 24 h post WSSV injection. **C** mRNA expression level of the WSSV late gene VP28 in hemocytes at 24 h post WSSV injection. **D** WSSV genome copy number was determined in shrimp pleopods at 24 h post WSSV injection. Shrimp treated with PBS or non-specific luciferase (Luc) dsRNA were used as control groups. Each bar represents the mean ± SD. Asterisks indicate statistically significant differences between the control group and *Lv*MDH1 or *Lv*MDH2 dsRNA treated group (*p < 0.05; **p < 0.01). Hcy: hemocytes. PL: pleopods

**Fig. 5:**
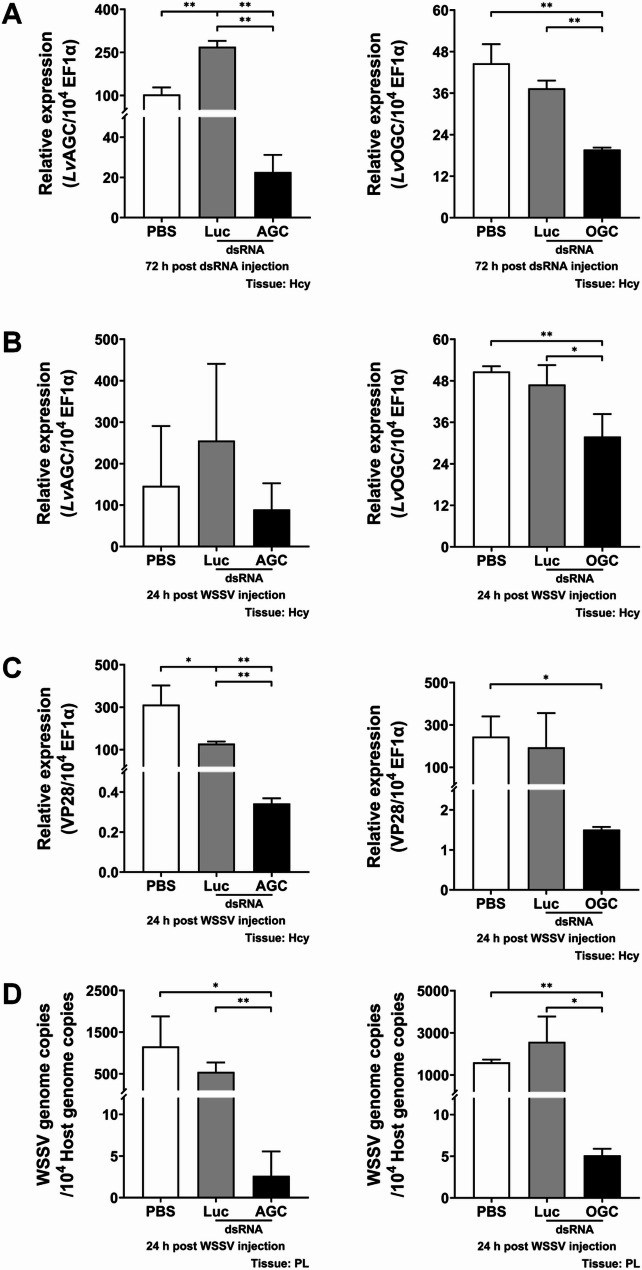
Effects of *Lv*AGC or *Lv*OGC silencing on WSSV replication. **A** mRNA expression levels of *Lv*AGC and *Lv*OGC in shrimp hemocytes at 72 h post dsRNA injection and before WSSV challenge. **B** mRNA expression levels of *Lv*AGC and *Lv*OGC in hemocytes at 24 h post WSSV injection. **C** mRNA expression level of the WSSV late gene VP28 in hemocytes at 24 h post WSSV injection. **D** WSSV genome copy number was determined in shrimp pleopods at 24 h post WSSV injection. shrimp treated with PBS or non-specific luciferase (Luc) dsRNA were used as control groups. Each bar represents the mean ± SD. Asterisks indicate statistically significant differences between the *Lv*AGC or *Lv*OGC dsRNA treated groups and the corresponding control group (*p < 0.05; **p < 0.01). Hcy: hemocytes. PL: pleopods

To assess the significance of the malate-aspartate shuttle in WSSV replication, we employed dsRNA-mediated *in vivo* gene silencing to knockdown expression of MAS-related genes and subsequently observe the effect of this treatment on WSSV replication. Shrimp were injected with respective dsRNAs 72 h before WSSV challenge, and hemocytes were collected at 24 h post WSSV injection for analysis. The mRNA expression levels of MAS-related genes, including *Lv*GOT1, *Lv*GOT2, *Lv*MDH1, *Lv*MDH2, *Lv*AGC and *Lv*OGC, were significantly reduced in their corresponding dsRNA-treated group at 72 h post dsRNA injection (Figs. 3A, 4A and 5A). Except for the treatment with dsRNA *Lv*AGC, the silencing effect also remained evident after WSSV challenge (Figs. 3B, 4B and 5B), demonstrating effective gene silencing throughout the WSSV infection. The silencing of MAS-related genes significantly suppressed mRNA expression of the WSSV late gene VP28 at 24 hpi compared to control groups treated with PBS or non-specific luciferase dsRNA (Figs. 3C, 4C and 5C). Furthermore, this silencing also significantly lowered the WSSV genome copy number at 24 hpi (Figs. 3D, 4D and 5D). These results suggested that the malate-aspartate shuttle plays an important role in WSSV replication.

### Replenishment with aspartate or α-ketoglutarate rescues WSSV replication impaired by silencing *Lv*GOT1, but not when *Lv*GOT2 is silenced

**Fig. 6:**
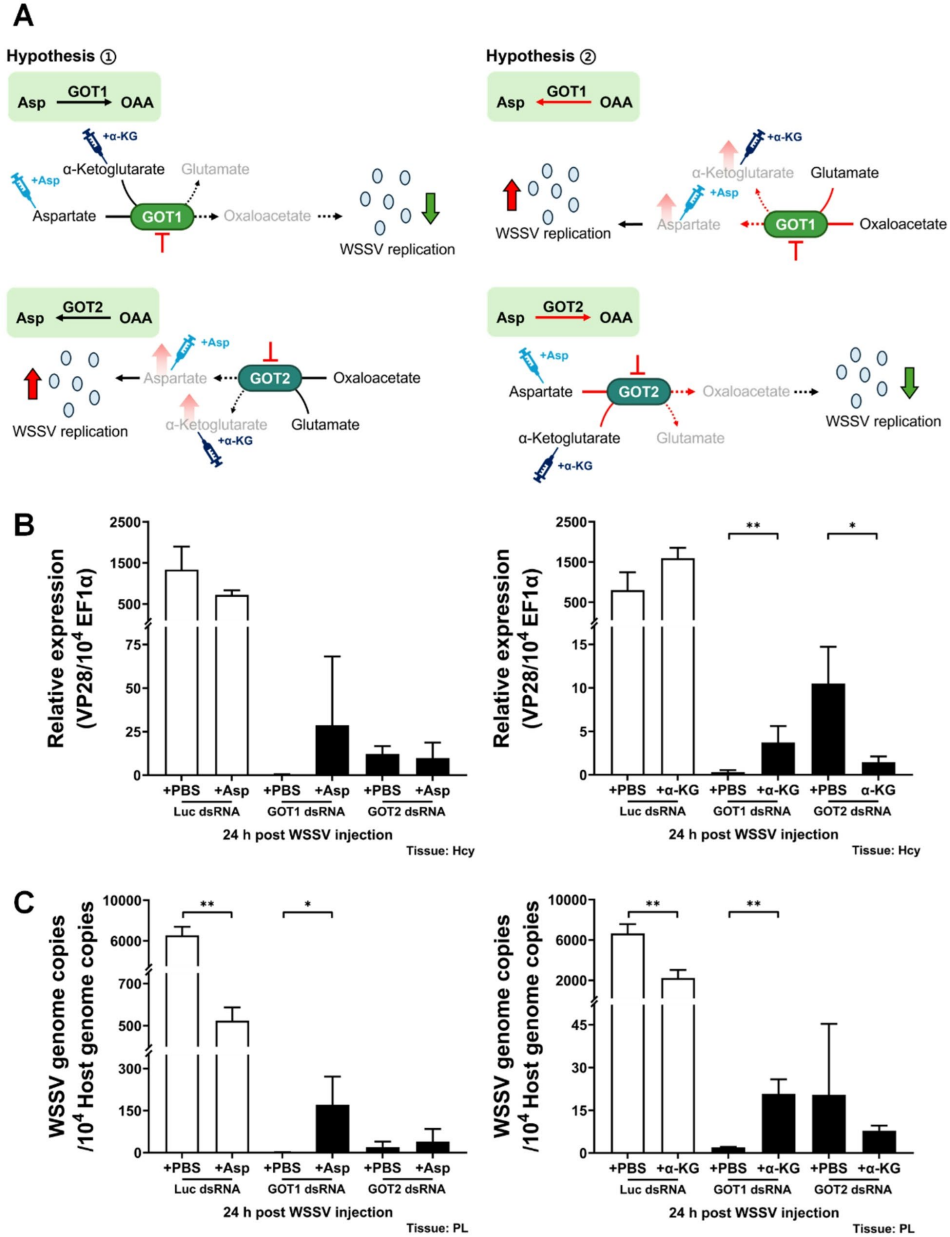
Effect of metabolite replenishment after silencing *Lv*GOT1 or *Lv*GOT2 during WSSV infection. **A** Schematic diagram of putative direction of reactions catalyzed by *Lv*GOT1 and *Lv*GOT2 during WSSV infection. In the experiment, shrimps were treated with *Lv*GOT1 or *Lv*GOT2 dsRNA at 72 h before WSSV challenge, and the indicated PBS/metabolite was injected at 2 h post WSSV injection. The dosage of aspartate was 15.6 µg per gram shrimp, and α-ketoglutarate was 530 µg per gram shrimp. **B** Effects of aspartate or α-ketoglutarate replenishment on mRNA expression level of WSSV late gene VP28 in *Lv*GOT1 or *Lv*GOT2-silenced shrimp hemocytes at 24 h post WSSV injection. **C** Effects of aspartate or α-ketoglutarate replenishment on WSSV genome copy number in *Lv*GOT1 or *Lv*GOT2-silenced shrimp pleopods at 24 h post WSSV injection. Groups treated with luciferase (Luc) dsRNA were used as non-specific silencing control groups. PBS was used as the replenishment control in all dsRNA-injected groups. Each bar represents the mean ± SD. Asterisks indicate statistically significant differences between PBS and the metabolite-replenished groups (*p < 0.05; **p < 0.01). Asp: aspartate. α-KG: α-ketoglutarate, OAA: oxaloacetate, Hcy: hemocytes, PL: pleopods

To elucidate the direction of metabolic flux in the malate-aspartate shuttle during WSSV infection, we used metabolite replenishment after silencing *Lv*GOT1 and *Lv*GOT2 in shrimp. Aspartate and α-ketoglutarate were selected for metabolite replenishment since they are both involved in the reversible GOT1/2-catalyzed reaction. Shrimps were treated with dsRNA at 72 h before WSSV challenge, and metabolites were injected at 2 h post WSSV challenge. Here, we propose two alternative hypotheses for the direction of the malate-aspartate shuttle during WSSV infection (Fig. 6A). Hypothesis 1 assumes the conventional direction of the malate-aspartate shuttle, whereby cytosolic *Lv*GOT1 will convert aspartate into oxaloacetate and simultaneously convert α-ketoglutarate into glutamate, while mitochondrial *Lv*GOT2 will convert oxaloacetate into aspartate and simultaneously convert glutamate into α-ketoglutarate. If the MAS operates in the direction suggested by hypothesis 1 during WSSV infection, then replenishment of α-ketoglutarate or aspartate will be unable to rescue the reduction of WSSV replication caused by silencing *Lv*GOT1, but it will be able to rescue WSSV replication caused by silencing *Lv*GOT2. Conversely in hypothesis 2, which assumes the reversed direction of the malate-aspartate shuttle, cytosolic *Lv*GOT1 will convert oxaloacetate into aspartate and simultaneously convert glutamate into α-ketoglutarate, while mitochondrial *Lv*GOT2 will convert aspartate into oxaloacetate and simultaneously convert α-ketoglutarate into glutamate. In the case of hypothesis 2, replenishment with aspartate or α-ketoglutarate should rescue the reduction of WSSV replication caused by silencing *Lv*GOT1, but should not be able to rescue WSSV replication if it is due to silencing *Lv*GOT2. We found that when *Lv*GOT1 was silenced, replenishment with aspartate had no significant effect on viral VP28 expression at 24 hpi, but replenishment of α-ketoglutarate significantly up-regulated viral VP28 expression compared to the GOT1 dsRNA-silenced, PBS-replenished group (Fig. 6B). In addition, replenishment with both aspartate and α-ketoglutarate increased the viral genome copy number at 24 hpi compared to the GOT1 dsRNA-silenced, PBS-replenished group (Fig. 6C). Meanwhile, when *Lv*GOT2 was silenced, replenishment with aspartate had no effect on viral VP28 expression or viral genome copy number (Figs. 6B and C), while replenishment of α-ketoglutarate left the viral genome copy number unaffected, and the viral VP28 expression level was actually reduced (Figs. 6B and C). Except for the lack of a significant effect on VP28 expression by aspartate after *Lv*GOT1 silencing, these results were entirely consistent with our hypothesis 2, indicating that WSSV triggers a reversal of the malate-aspartate shuttle during infection.

### Replenishment with oxaloacetate rescues WSSV replication impaired by silencing *Lv*MDH1, but not when *Lv*MDH2 is silenced

**Fig. 7:**
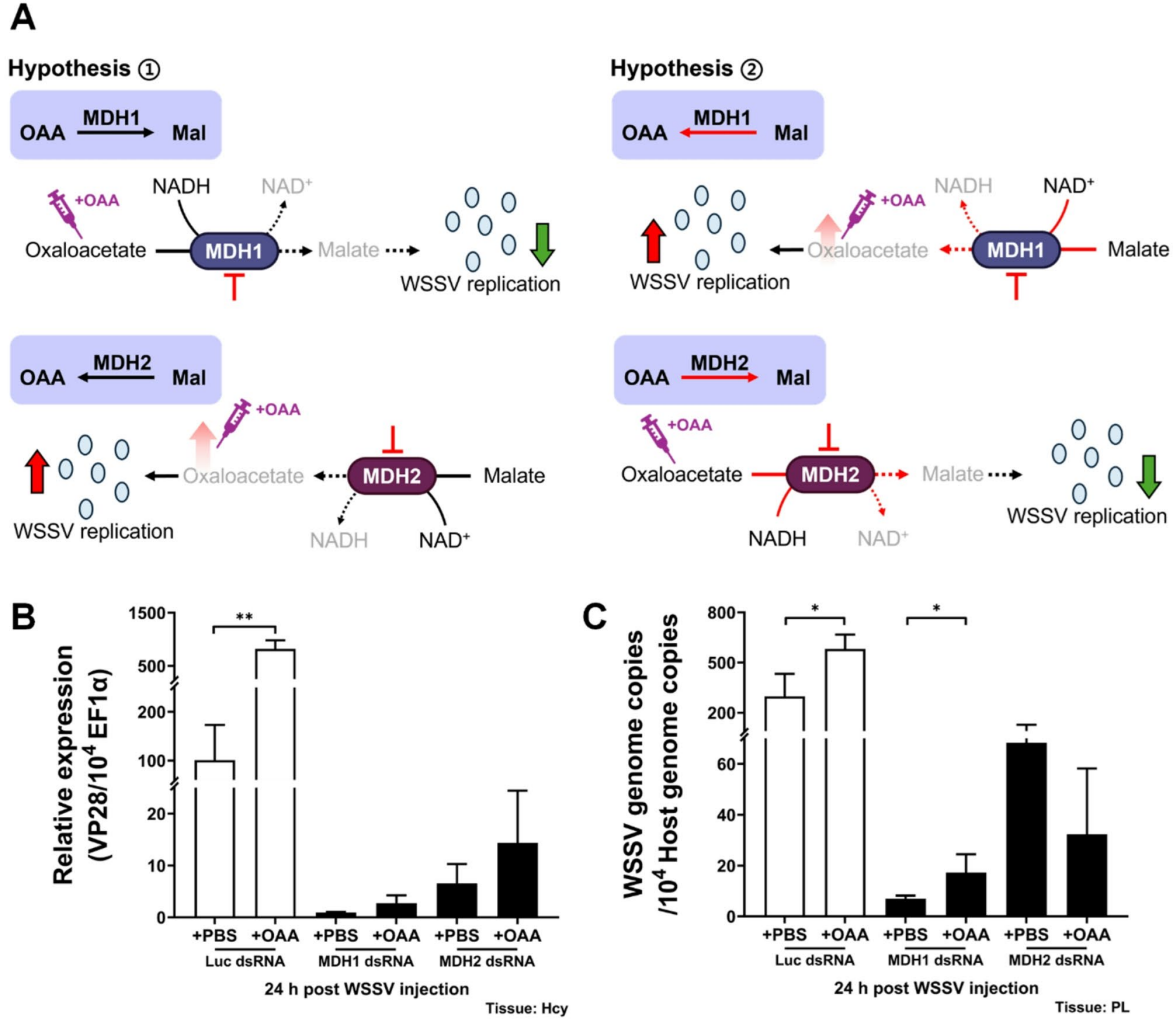
Effect of metabolite replenishment after silencing *Lv*MDH1 or *Lv*MDH2 during WSSV infection. **A** Schematic diagram of putative direction of reactions catalyzed by *Lv*MDH1 and *Lv*MDH2 during WSSV infection. In the experiment, shrimps were treated with *Lv*MDH1 or *Lv*MDH2 dsRNA at 72 h before WSSV challenge, and PBS/oxaloacetate were injected at 2 h post WSSV infection. The dosage of oxaloacetate was 23.4 µg per gram shrimp. **B** Effects of oxaloacetate replenishment on mRNA expression level of WSSV late gene VP28 in *Lv*MDH1 or *Lv*MDH2-silenced shrimp hemocytes at 24 h post WSSV injection. **C** Effects of oxaloacetate replenishment on WSSV genome copy number in *Lv*MDH1 or *Lv*MDH2-silenced shrimp pleopods at 24 h post WSSV injection. Groups treated with non-specific luciferase (Luc) dsRNA were used as control groups. PBS was used as the replenishment control in all dsRNA-injected groups. Each bar represents the mean ± SD. Asterisks indicate statistically significant differences between PBS and a metabolite-replenished group (*p < 0.05; **p < 0.01). OAA: oxaloacetate, Mal: malate, Hcy: hemocytes, PL: pleopods

To further validate our hypothesis about the direction of malate-aspartate shuttle during WSSV infection, we also conducted oxaloacetate replenishment after silencing *Lv*MDH1 and *Lv*MDH2 in shrimp. In Fig. 7A, hypothesis 1 illustrates the conventional direction of the malate-aspartate shuttle. Cytosolic *Lv*MDH1 will convert oxaloacetate into malate in conjunction with the oxidation of NADH to NAD^+^, while mitochondrial *Lv*MDH2 will convert malate into oxaloacetate in conjunction with the reduction of NAD^+^ to NADH. If the MAS operates in the direction suggested by hypothesis 1 during WSSV infection, replenishment with oxaloacetate will be unable to rescue the reduction of WSSV replication caused by silencing *Lv*MDH1, but it should be able to rescue viral replication when *Lv*MDH2 is silenced. In hypothesis 2, which assumes the reversed direction of the malate-aspartate shuttle, cytosolic *Lv*MDH1 will convert malate into oxaloacetate in conjunction with the reduction of NAD^+^ to NADH, while mitochondrial *Lv*MDH2 will convert oxaloacetate into malate in conjunction with the oxidation of NADH to NAD^+^. In the case of hypothesis 2, replenishment with oxaloacetate should be able to rescue the reduction of WSSV replication when it is caused by silencing *Lv*MDH1 but not when it is caused by silencing *Lv*MDH2. Results demonstrated that, in the luciferase dsRNA control group, significant increase in both viral VP28 expression and viral genome copy number were observed after oxaloacetate supplementation compared to the PBS vehicle alone (Figs. 7B and C). However, when either *Lv*MDH1 or *Lv*MDH2 was silenced, replenishment with oxaloacetate showed no significant changes in viral VP28 expression (Fig. 7B). On the other hand, replenishment with oxaloacetate significantly increased viral genome copy number compared to the PBS control when *Lv*MDH1 was silenced (Fig. 7C). Combined with the findings from Fig. 6, we concluded that WSSV triggers reversed operation of the malate-aspartate shuttle during infection.

## Discussion

During infection, viruses hijack and rewire host cell metabolism to generate building blocks and energy necessary for viral replication [37–39]. The crustacean DNA virus White Spot Syndrome Virus (WSSV) induces extensive metabolic reprogramming in host cells [26]. In this study, we reveal the critical role of the malate-aspartate shuttle (MAS) in shrimp during WSSV replication and provide initial insights into how MAS is modulated during WSSV infection. MAS serves as a crucial mechanism in which cells transfer cytosolic NADH into mitochondria, thereby maintaining cellular redox homeostasis [5]. Several studies have demonstrated that MAS-mediated NADH shuttling and cytosolic NAD⁺ regeneration support the high glycolytic demand of proliferating cells [14, 16, 40]. MAS-related genes are highly expressed in various types of cancers, and blocking MAS has been shown to cause redox imbalance (reductive stress) and metabolic disorder, ultimately suppressing cancer cell growth [12, 13, 15, 16, 18–20, 41]. Likewise, upregulation of MAS components, particularly the aminotransferases GOT1 and GOT2, has been observed during viral infections. MAS inhibition impaired viral replication, further highlighting its importance [21–24]. Consistent with these findings, our study demonstrates that there is significant upregulation of MAS-related genes at 12 hpi, including *Lv*GOT1, *Lv*GOT2, *Lv*MDH1, *Lv*AGC, and *Lv*OGC (Fig. 2), suggesting that MAS is activated at the viral genome replication stage to support both NADH shuttling, the transfer of reducing equivalents generated from WSSV-induced glycolysis, and glutamine-driven anaplerosis. *In vivo* dsRNA-mediated gene silencing showed that targeting any of these MAS components caused significant decreases in both WSSV gene expression and viral genome copy number (Figs. 3, 4 and 5), verifying the critical role of MAS in WSSV replication. Since GOT1 and GOT2 are also key enzymes in glutamine metabolism, these results are also consistent with our previous studies, which showed that WSSV replication is highly dependent on glutamine metabolism. We also note that the functional domain of transporter AGC contains a calcium-binding EF-hand motif, implying that the activity of AGC may be regulated by intracellular Ca²⁺ levels. Some studies have revealed that elevated Ca²⁺ concentrations can promote AGC activity [42, 43], while WSSV infection has been reported to induce an increase in intracellular Ca²⁺ [44, 45], which may activate *Lv*AGC and further stimulate MAS operation.

**Fig. 8:**
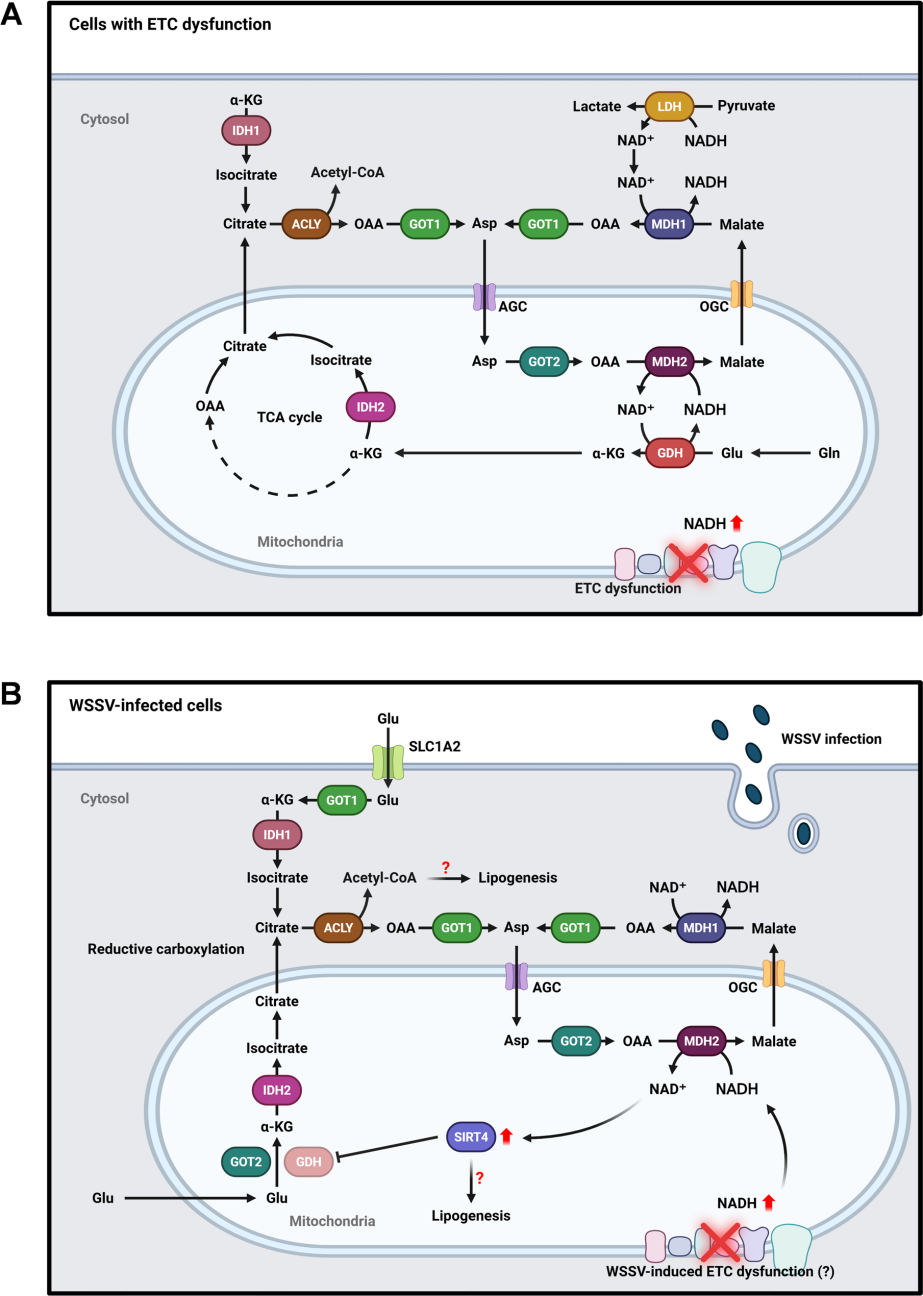
Metabolism similarities between cells with ETC dysfunction and WSSV-infected cells. **A** Schematic diagram illustrating malate-aspartate shuttle (MAS) in a cell with dysfunctional electron transport chain (ETC). Under conditions of ETC deficiency, GOT1 catalyzes the reverse reaction to produce aspartate, while aspartate serves as a substrate for the reverse activities of GOT2 and MDH2 in regenerating NAD+ to support GDH-mediated glutamine anaplerosis. This figure was adapted from Birsoy et al. (2015) [52] and Altea-Manzano et al. (2022) [53]. **B** WSSV infection can lead to a loss of mitochondrial membrane potential (MMP) and reduced ATP production, both of which are hallmarks of ETC dysfunction. We speculate that WSSV induces ETC dysfunction, leading to an excessive accumulation of NADH. In response, the host may activate reversed operation of MAS to reduce the elevated levels of NADH in the mitochondria. In addition, the NAD+ produced may be used to fuel lipogenesis instead of supporting GDH activity. GOT1/2, glutamate-oxaloacetate transaminase 1/2. MDH1/2, malate dehydrogenase 1/2. AGC, aspartate-glutamate carrier. OGC, 2-oxoglutarate carrier. GAPDH, glyceraldehyde 3-phosphate dehydrogenase. SLC1A2, solute carrier family 1 member 2. ACLY, ATP citrate lyase. IDH1/2, isocitrate dehydrogenase 1/2. SIRT4, sirtuin 4. GDH, glutamate dehydrogenase. Glu, glutamate.

Supplementation with the metabolites aspartate and α-ketoglutarate (α-KG) has been shown to rescue cancer cell proliferation by GOT2 inhibition [16], and another study reported that supplementation with the GOT1 downstream metabolite oxaloacetate could partially rescue viability of GOT1-knockout cells [46]. In our study, supplementation with aspartate or α-ketoglutarate failed to rescue WSSV gene expression and genome copy number following silencing *Lv*GOT2, while only α-ketoglutarate was able to rescue the viral replication that was impaired by silencing *Lv*GOT1 (Fig. 6). A similar pattern was observed in oxaloacetate supplementation following *Lv*MDH1 or *Lv*MDH2 silencing—oxaloacetate partially restored viral replication after silencing *Lv*MDH1 but not *Lv*MDH2 (Fig. 7). According to our hypotheses regarding MAS direction during WSSV infection (Figs. 6A and 7A), we therefore propose that WSSV triggers reversal of the malate-aspartate shuttle during WSSV infection. Under normal conditions, the proton gradient across the mitochondrial inner membrane drives the MAS unidirectionally, transferring NADH from cytosol to mitochondria for ETC and subsequent ATP production [5]. However, MAS might operate in the reversed direction under these three conditions: (1) loss of mitochondrial membrane potential, (2) low mitochondrial NAD⁺/NADH ratio, and (3) high cytosolic NAD⁺/NADH ratio [47]. Loss of mitochondrial membrane potential is the key consequence of a dysfunctional electron transport chain [48]. Under these conditions, ATP production is affected and NADH accumulation inhibits the NADH-generating enzyme in the TCA cycle, ultimately shutting down the TCA cycle altogether [49, 50]. Gottlieb et al. (2003) further showed that this loss of mitochondrial membrane protein induces cytochrome c release from mitochondria, triggering caspase-mediated apoptosis [51]. Interestingly, Chen et al. (2011) also reported a loss of mitochondrial membrane potential and an elevated ADP/ATP ratio at the late stage of WSSV infection [28]. Taken together, the WSSV-induced reversed MAS observed in the present study and the findings of Chen et al. (2011) suggest that the ETC might be impaired during WSSV infection [28]. In proliferating cells, the ETC primarily supports the MAS to produce aspartate, an essential metabolite required for nucleotide and amino acid biosynthesis [52]. When the ETC is inhibited, reductive glutamine metabolism and pyruvate metabolism via GOT1 will be activated to compensate for the deficiency of aspartate [52]. Moreover, in ETC-deficient cells, GOT2 and MDH2 reverse their reaction, using aspartate to oxidize mitochondrial NADH to NAD^+^. This reverse flux sustains the NAD^+^-dependent activity of the mitochondrial glutamate dehydrogenase (GDH), thus allowing the glutamine-driven anaplerosis that supports cell viability [53]. Taking into account these studies of ETC dysfunction, we propose that WSSV infection triggers a similar metabolic reprogramming to that seen in ETC-impaired cells. This would serve the following purposes (Fig. 8): (1) WSSV-induced reversed MAS could regenerate NAD^+^ to prevent TCA shut down. (2) The aspartate from WSSV-induced oxidative glutamine metabolism or reductive carboxylation could serve as a critical precursor of the building blocks necessary for virus replication.

By using stable-isotope labeled U-¹³C glutamine tracking, we previously revealed that increased levels of glutamate and α-ketoglutarate were seen at 24 hpi of WSSV infection, while 1-¹³C-glutamine tracking further revealed significant increases in the citrate derived from reductive carboxylation [32]. The mRNA expression level of *Lv*IDH1, a key enzyme involved in reductive carboxylation, was also upregulated at 24 hpi [32]. These findings suggest that WSSV continues to induce reductive glutamine metabolism at the late stage of infection. However, despite a significant increase in *Lv*GDH mRNA expression, its enzymatic activity was notably decreased at 24 hpi [33], which was inconsistent with the Altea-Manzano et al. (2022) [53] study that MDH2-mediated oxidation of NADH to NAD^+^ supports GDH activity. However, it is possible that the reduction in *Lv*GDH activity may be attributed to its inhibition by *Lv*SIRT4 in WSSV-infected cells: Tan et al. (2024) reported that there was a significant upregulation of *Lv*SIRT4 mRNA expression at 24 hpi, and that *Lv*GDH activity could be restored by silencing *Lv*SIRT4, suggesting that *Lv*GDH activity is negatively regulated by *Lv*SIRT4 [54]. Interestingly, both *Lv*SIRT4 and *Lv*GDH are NAD⁺-dependent enzymes. Thus, we speculate that NAD⁺ generated through *Lv*MDH2-mediated oxidation of mitochondrial NADH preferentially supports *Lv*SIRT4 activation rather than *Lv*GDH activity at the late stage of WSSV infection. Since SIRT4 is a key regulator of lipogenesis, its activation may promote lipogenesis to facilitate virion morphogenesis [55, 56]. Additionally, WSSV-induced reductive glutamine metabolism supplies citrate to the cytosol, where it may serve as a substrate for ATP citrate lyase to generate acetyl-CoA, ultimately promoting lipogenesis during viral replication [32]. Significant upregulation of the glutamate transporter *Lv*SLC1A2 (solute carrier family 1 member 2) has also been observed at 24 hpi, indicating that WSSV-infected cells preferentially utilize glutamate rather than glutamine as a carbon source [33]. The conversion of glutamate to α-ketoglutarate is likely mediated by *Lv*GOT1/2 instead of *Lv*GDH, further supporting the occurrence of reductive carboxylation at the late stage of WSSV infection.

This study raises an important question regarding the timing of the reversed MAS activation during WSSV replication. Determining the precise stages at which this pathway is engaged could provide key insights into its functional role in supporting viral propagation. In our previous work, we employed stable isotope-labeled metabolites to perform metabolic flux tracking in shrimp [27, 29, 32]. This approach can be extended to delineate how MAS flux is redirected at defined infection phases — particularly during the early infection stage, the viral genome replication stage (12 hpi), and the late stage (24 hpi). Further investigation is required to elucidate the molecular mechanisms by which WSSV regulates this process. Although most WSSV ORFs show no significant homology to known proteins, a subset shares similarities with shrimp host genes [57]. Notably, the WSSV genome encodes enzymes involved in *de novo* nucleotide biosynthesis, such as thymidine kinase, ribonucleotide reductase, and thymidylate synthase [57]. This observation raises the possibility that certain WSSV ORFs may have MAS-like enzymatic activities that facilitate metabolic reprogramming during infection. Alternatively, WSSV proteins may directly interact with the host enzymes involved in these pathways, as exemplified by WSSV004’s interaction with *Lv*LDH [58]. We are currently conducting a yeast two-hybrid screening to identify additional WSSV proteins that interact with MAS-related host factors. Finally, the impact of WSSV-induced MAS reversal on the cellular NADH/NAD⁺ ratio and the resulting oxidative stress remains an open question that warrants further exploration.

## Conclusion

In conclusion, our study observes the reversal of the malate-aspartate shuttle (MAS) in *Litopenaeus vannamei* during WSSV replication and provides further evidence that WSSV infection induces a metabolic regulation that is similar to the one observed in cells with ETC dysfunction (Fig. 8). By means of this regulation, infected cells can generate specific metabolites (e.g., aspartate) required for virus replication or to prevent TCA shut down throughout virus replication. Our findings underscore the need for further investigation into MAS’s role in WSSV infection.

## Supplementary Information

Supplementary Material 1.

Supplementary Material 2.

## Data Availability

The datasets used and/or analyzed during the current study are available from the corresponding author on reasonable request.

## Abbreviations

α-KG: α-ketoglutarate
ADP: Adenosine diphosphate
AGC: Aspartate-glutamate carrier
Asp: Aspartate
ATP: Adenosine triphosphate
ETC: Electron transport chain
Glu: Glutamate
GOT: Glutamate-oxaloacetate transaminase
Hcy: Hemocytes
NAD: Nicotinamide adenine dinucleotide
Mal: Malate
MAS: Malate-aspartate shuttle
MDH: Malate dehydrogenase
Luc: Luciferase
Lv: Litopenaeus Vannamei
OAA: Oxaloacetate
OGC: Oxoglutarate carrier
ORF: Open reading frame
PBS: Phosphate Buffered Saline
PL: Pleopods
SIRT4: Sirtuin 4
TCA: Tricarboxylic Acid
WSSV: White spot syndrome virus
