# Supplementary material for: WSSV-induced reversal of the malate-aspartate shuttle facilitates viral replication in shrimp hemocytes
Source: Cell Commun Signal. 2025 Nov 26;23:507. doi: 10.1186/s12964-025-02506-3 (PMC12659559; doi:10.1186/s12964-025-02506-3)
Supplement: Supplementary file 1 — Supplementary Material 1. [file 12964_2025_2506_MOESM1_ESM.docx]

**Additional file 1**

**Figure S1. Silencing efficiency of *Lv*GOT1 & *Lv*GOT2 dsRNA in the aspartate and α-ketoglutarate replenishment experiment.** This data serves as supplementary information to Figure 6 in the main text. Shrimp were treated with *Lv*GOT1 or *Lv*GOT2 dsRNA at 72 hours before WSSV challenge, and the indicated PBS/metabolite was injected at 2 hours post WSSV infection. Hemocytes were collected at 24 hpi for mRNA expression analysis of (A) *Lv*GOT1 and (B) *Lv*GOT2. Groups treated with luciferase (Luc) dsRNA were used as non-specific silencing control groups. Each bar represents the mean ± SD. Asterisks indicate statistically significant differences between groups (*p < 0.05; **p < 0.01). Asp: aspartate. α-KG: α-ketoglutarate, Hcy: hemocytes.

**Figure S2. Silencing efficiency of *Lv*MDH1 & *Lv*MDH2 dsRNA in the oxaloacetate replenishment experiment.** This data serves as supplementary information to Figure 7 in the main text. Shrimp were treated with *Lv*MDH1 or *Lv*MDH2 dsRNA at 72 hours before WSSV challenge, and the indicated PBS/metabolite was injected at 2 hours post WSSV infection. Hemocytes were collected at 24 hpi for mRNA expression analysis of (A) *Lv*MDH1 and (B) *Lv*MDH2. Groups treated with luciferase (Luc) dsRNA were used as non-specific silencing control groups. Each bar represents the mean ± SD. Asterisks indicate statistically significant differences between groups (*p < 0.05; **p < 0.01). OAA: oxaloacetate, Hcy: hemocytes
